# Positioning equity at the core of European dementia research: a pan-European co-produced perspective from the PANEUCARE consortium

**DOI:** 10.3389/fmed.2026.1828363

**Published:** 2026-08-31

**Authors:** Maria Isabel Cardona, Clarissa Giebel, Anthony Scerri, Anthea Innes, Ninoslav Mimica, Osman Kučuk, T. Rune Nielsen, Jūratė Macijauskienė, Birute Bartkevičiūtė, Carolien Smits, Carmel Geoghegan, Soraya Moradi-Bachiller, Anne Marie Rokstad, Iva Holmerova, Elżbieta Trypka, Ágnes Egervári, Raluca Sfetcu, Marija Taneska, Péter Hegedűs, Iryna Shevchenko, Svetlana Iloski, Thanos Chatzikostopoulos, Jochen René Thyrian

**Affiliations:** 1 German Center for Neurodegenerative Diseases (DZNE), Rostock/Greifswald, Germany; 2 Department of Primary Care and Mental Health, University of Liverpool, Liverpool, United Kingdom; 3 NIHR Applied Research Collaboration North West Coast, Liverpool, United Kingdom; 4 Department of Nursing, University of Malta, Msida, Malta; 5 University of the Highlands and Islands (UHI), Inverness, Scotland, United Kingdom; 6 University of Zagreb, University Psychiatric Hospital Vrapče, Zagreb, Croatia; 7 Centre for Dementia in Sarajevo, Sarajevo, Bosnia and Herzegovina; 8 Danish Dementia Research Centre, Copenhagen University Hospital—Rigshospitalet, Copenhagen, Denmark; 9 Department of Geriatrics, Lithuanian University of Health Sciences (LSMU) Kaunas Hospital, Kaunas, Lithuania; 10 Dutch Centre of Expertise on Health Disparities, Utrecht, Netherlands; 11 Dementia Ireland Empowering Communities, Connemara, Ireland; 12 Alzheimer Europe, Luxemburg, Luxembourg; 13 Norwegian National Centre for Ageing and Health, Vestfold Hospital Trust, Tønsberg, Norway; 14 Charles University, Centre of Expertise in Longevity and Long-term Care, Praha, Czechia; 15 Wrocław Medical University, Wrocław, Poland; 16 Boldog Gizella Alapítvány, Biatorbágy, Hungary; 17 Social Cluster Association, Budapest, Hungary; 18 National School of Public Health, Management and Professional Development Bucharest, Department of Psychology and Educational Sciences, Spiru Haret University, Bucharest, Romania; 19 Alzheimer’s Society UK, London, United Kingdom; 20 Nezabutni Charitable Foundation, Kyiv, Ukraine; 21 Institute for Alzheimer's Disease and Neuroscience (IAN), Skopje, North Macedonia; 22 Greek Association of Alzheimer’s Disease and Related Disorders, Thessaloniki, Greece; 23 Institute for Community Medicine, University Medicine Greifswald, Greifswald, Germany

**Keywords:** co-production, dementia care, equity, European public health, pan-European research

## Abstract

Dementia care across Europe is characterized by substantial regional disparities in research participation, funding, and care practices, which challenge the development of equitable and inclusive research agendas. The PANEUCARE consortium adopted a pan-European, co-produced approach that integrates professional expertise with the perspectives of people living with dementia and caregivers to identify priorities for a more equitable dementia research landscape. Insights were generated through two workshops with dementia professionals and consultation groups involving people living with dementia and caregivers, followed by deliberative synthesis to explore common challenges, regional barriers, and shared research priorities. Participants highlighted persistent issues including workforce shortages, delayed diagnosis, fragmented care pathways, caregiver burden, and the underrepresentation of Southern and Eastern European contexts in research. At the same time, examples of local innovation, such as community-based services, cross-sector collaboration, and the use of digital tools, demonstrated context-specific resilience and opportunities for cross-regional learning. Priority areas for future research included strengthening cross-regional collaboration, addressing workforce development, improving culturally sensitive and post-diagnostic care, enhancing support for caregivers, and promoting more inclusive participation in research. These insights informed the development of the EQUITABLE framework (Equity, Quality, Urgency, Involvement, Transdisciplinary collaboration, Adaptability, Budget-consciousness, Linguistic and cultural sensitivity, and Engagement), which provides actionable guidance for designing inclusive and context-sensitive dementia research across Europe. By bringing together professional expertise and lived experience, PANEUCARE highlights how Europe's diversity in dementia care can be leveraged as a resource for shared learning and policy development, supporting a more equitable, sustainable, and responsive European dementia research landscape.

## 1 Introduction

Dementia affects an estimated 55 million worldwide (1). As Europe's population ages, prevalence is expected to increase, placing pressure on health and social care systems (1, 2). It affects cognitive, behavioural, and functional capacities and generates emotional, social, and economic impacts for families and health systems (1).

Across Europe, dementia care research and policy have advanced considerably, with several countries developing national strategies, research frameworks, and funding programmes (3). However, important regional disparities remain in research visibility, participation, and systemic capacity. Southern and Eastern European countries, while contributing valuable perspectives, are still underrepresented in collaborative projects, funding opportunities, and policy discussions (3). These imbalances may limit European dementia research by overlooking contextual knowledge, innovations, and care needs (4, 5). As a result, the European dementia research landscape continues to be described as fragmented, with uneven research capacity, disparities in funding opportunities, and limited representation of diverse care realities (4–6).

Healthcare coverage across the countries represented in PANEUCARE varies in its organisation, financing, scope of benefits, and degree of integration between health and social care (7). Although most European countries provide statutory or publicly funded healthcare coverage, differences in co-payments, eligibility criteria, referral pathways, availability of specialist services, and coverage of long-term and post-diagnostic support can substantially affect access to dementia care (7). Formal entitlement to healthcare does not therefore necessarily translate into timely or equitable access to diagnosis, specialist assessment, treatment, or community-based support (6). Migrants may also experience additional barriers to accessing dementia-related services. Administrative requirements, language barriers, limited knowledge of available services, cultural differences, and difficulties navigating healthcare systems may restrict effective access to dementia diagnosis and support (8, 9).

Addressing these gaps requires more than documenting differences between European countries. It requires understanding which challenges are shared across Europe, which are context-specific, and how professional expertise and lived experience together can inform more equitable and inclusive dementia research. Although previous studies have described variations in dementia care, research capacity, and access to services across Europe (3), there remains a need to understand how shared research priorities can be identified while recognising the diversity of European dementia care systems. Integrating professional expertise with the perspectives of people living with dementia and caregivers can strengthen the identification of shared priorities and the co-production of research frameworks that are responsive to different health and care contexts (10, 11). This Perspective addresses this gap by synthesising professional and lived-experience perspectives into a set of guiding principles to support equitable, inclusive, and context-sensitive dementia research across diverse European contexts.

In this context, the PANEUCARE (Pan-European Knowledge Exchange on Dementia Care and Research) consortium was established within the EU Joint Programme on Neurodegenerative Disease Research (JPND). Drawing on insights from professionals, people living with dementia, and caregivers across Europe, this Perspective first examines regional diversity and lived experiences of dementia care, then identifies shared priorities for future research, and finally synthesises these findings into the EQUITABLE framework, providing guiding principles for more equitable, inclusive and context-sensitive dementia research across Europe.

## 2 Methods

The PANEUCARE consortium brought together 22 professionals with expertise in dementia research, practice, and policy from 19 countries (Bosnia and Herzegovina, Croatia, Czech Republic, Denmark, Germany, Greece, Hungary, Ireland, Lithuania, Luxembourg, Malta, Netherlands, North Macedonia, Norway, Poland, Romania, Ukraine, the United Kingdom, and Canada). Consortium members were invited based on their relevant expertise and with the aim of bringing together complementary professional perspectives from different European regions and supporting geographical representation from underrepresented Southern and Eastern European countries.

To incorporate lived-experience perspectives, consultation groups were conducted with the European Working Group of People with Dementia (EWGPWD) and the European Dementia Carers Working Group (EDCWG), coordinated by Alzheimer Europe. In total, 23 individuals participated, including people with dementia and caregivers from multiple European countries. Participants were members of these established European advisory groups, ensuring the direct involvement of people with lived experience in identifying research priorities.

PANEUCARE adopted a structured three-phase consultation process. The first workshop (Bonn, 22–23 April 2,024) gathered professionals to present country-specific dementia care profiles and discuss national challenges, examples of good practice, and research needs using a common discussion framework. Consultation groups with people living with dementia and caregivers were subsequently held in Brussels (11 December 2024), exploring experiences of dementia care, barriers to accessing services, priorities for future research, and examples of good practice. A second workshop (Berlin, 6–7 March 2025) integrated findings from the professional workshop and consultation groups to refine common themes and discuss priorities for future dementia research.

Data generated during the first workshop were analysed using qualitative content analysis following the principles described by Mayring (12). Workshop notes, country profiles and presentation materials were reviewed manually to identify recurring challenges, examples of good practice and emerging research priorities. Relevant text segments were assigned descriptive categories, which informed the development of the discussion guide for the consultation groups with people living with dementia and caregivers. Insights from these consultation groups were then used to refine and expand the preliminary thematic categories. During the second workshop, these integrated findings were discussed with consortium members and supported by a structured electronic ranking exercise to inform the identification of shared research priorities and the development of the EQUITABLE framework. The ranking exercise served to facilitate discussion and reflection on the emerging priorities but was not intended to establish a hierarchical ordering of themes or a formal consensus.

## 3 Results

### 3.1 Regional diversity in dementia research

Insights from the first PANEUCARE workshop showed that European countries face common dementia care challenges. These include demographic ageing, rising dementia prevalence, limited access to specialised care and support, workforce shortages, delayed diagnoses, caregiver burden, and insufficient dementia-specific training. Two systemic issues were prominent: limited coordination between health and social care systems and a persistent gap between policy development and practical implementation.

These challenges manifest differently across regions, reflecting Europe's socio-economic and political diversity. Participants from Southern and Eastern Europe emphasised structural barriers such as limited financial resources, lower political prioritisation of dementia, and the absence or incomplete implementation of national dementia strategies. At the same time, locally adapted responses and community-driven initiatives in these regions are often underrepresented in European policy discussions.

Participants from Western and Northern Europe highlighted somewhat different priorities, including improving service accessibility and coordination, strengthening home-based care models, addressing rural and culturally appropriate care needs, and implementing quality standards in dementia services.

These regional groupings represent recurring patterns identified during the consultation process rather than uniform national profiles. Considerable variation exists both within and across European countries, and the distinctions presented here are intended to highlight common challenges and examples of good practice rather than to suggest homogeneous regional experiences.

Beyond these challenges, the PANEUCARE discussions identified a range of good practices across participating countries, including support services, caregiver support, awareness and advocacy initiatives, information dissemination, professional training, home-care initiatives, and dementia-inclusive community approaches. While many of these practices were reported across different European contexts, their organisation and implementation varied according to local health and social care structures. Community-based initiatives, public awareness campaigns, caregiver support services, home-care programmes, and volunteer-led activities were highlighted particularly in Southern and Eastern Europe. These initiatives demonstrate socially grounded responses despite structural constraints. In Western and Northern European countries, examples included dementia case management systems, integrated health and social care data infrastructures, person-centred care approaches, dementia care mapping, and specialised research infrastructures. These examples illustrate how shared challenges can generate different locally adapted responses and opportunities for mutual learning across European contexts.

### 3.2 Lived experience in dementia care and research

Consultation groups with people living with dementia and caregivers provided direct insight into care experiences and priorities across Europe. Participants described barriers to accessing care and support, particularly in rural and remote regions, socioeconomically disadvantaged communities, areas with shortages of healthcare professionals, and communities facing linguistic, cultural, or transport-related barriers. Delayed diagnoses, unclear care pathways, and fragmented services were commonly reported. Even in countries with national dementia strategies, many reported difficulties navigating services after diagnosis. Access also differed between primary care and specialist services. General practitioners or primary care physicians were generally more geographically accessible and often represented the first point of contact; however, limited consultation time, variable dementia-specific training, and complex referral pathways could delay recognition and diagnosis. Access to neurologists, geriatricians, psychiatrists, specialist nurses, and multidisciplinary memory clinics was often more restricted, particularly in rural and under-resourced settings, and could involve longer waiting times and considerable travel. Consequently, the availability and coordination of both primary and specialist care influenced the timeliness and continuity of dementia assessment and support.

Quality of care emerged as a major concern. Participants reported experiences of stigma, misinformation, and limited understanding of dementia among healthcare professionals, reinforcing the need for dementia-specific training across all levels of care. People living with dementia also emphasised the importance of meaningful participation in care decisions, including support for advance care planning and involvement in shaping their own care pathways.

Caregivers echoed many of these concerns while highlighting additional challenges, including long waiting times for diagnosis in some regions, limited access to specialists in rural areas, fragmented coordination between health and social care services, and insufficient support for young-onset dementia. Both people with dementia and caregivers emphasised the need for stronger emotional, practical, and financial support mechanisms within healthcare systems.

Both groups also expressed interest in research participation but identified barriers such as limited information about opportunities and insufficiently inclusive mechanisms. Examples from the Netherlands demonstrated the potential of participatory research models in which people living with dementia contribute as co-researchers. During prioritisation discussions, people living with dementia emphasised care quality, timely diagnosis, and access to reliable information, while caregivers prioritised home-care support, trained professionals, and person-centred care. Both groups highlighted the importance of dementia-inclusive communities and accessible technological solutions adapted to diverse contexts.

The experiences described by people living with dementia and caregivers were closely aligned with the structural patterns identified during the professional consultations. Challenges such as delayed diagnosis, fragmented care pathways, limited access to specialist services, and difficulties navigating support systems reflected many of the system-level issues identified across participating countries. Together, these findings indicate how structural characteristics of dementia care systems are reflected in the everyday experiences of people living with dementia and caregivers across diverse European contexts.

### 3.3 System-level priorities for future dementia care

Building on these findings, the second PANEUCARE workshop synthesised expert and consultation-group insights to identify priorities for future dementia care research and policy. Rather than ranking individual topics, participants highlighted a set of interconnected priorities reflecting shared challenges across European regions while recognising contextual differences.

One priority concerns **workforce capacity**. In European dementia care systems, particularly in Southern and Eastern regions and the UK, shortages and emigration of trained dementia care professionals represent a bottleneck. These shortages limit access to services, reduce care quality and continuity, and increase regional disparities and staff burden (13, 14). Participants emphasised the need for innovative training programmes, improved working conditions, and stronger career incentives to attract and retain professionals in dementia care. Examples such as e-learning platforms in Norway and Denmark illustrate scalable approaches to workforce development. Sustainable workforce strategies should address training, retention, and equitable regional distribution (14, 15).

Another priority relates to the **organisation and continuity of dementia care**. Fragmentation between medical and social care services often disrupts coordinated care across sectors and stages of the disease trajectory. Emerging approaches, including care coordinators and digital coordination platforms (e.g., Germany's Dementia Networks, Denmark's primary care dementia coordinators, Malta's dementia intervention team, and the Netherlands’ Social Approach), illustrate pathways toward more integrated and person-centred care (16, 17). Longitudinal care models should respond to evolving needs from diagnosis to palliative stages and address cognitive, physical, social, and emotional dimensions affecting patients and caregivers (18). However, many systems still focus on isolated stages of care, creating gaps and inequities across the disease trajectory (19). The absence of shared European frameworks further limits comparability, scalability, and equitable access to integrated care models (20, 21).

**Post-diagnostic support and caregiver inclusion** also emerged as key priorities. Research should prioritise interventions that equip family and professional caregivers with information, skills, and emotional support. Caregiver inclusion is essential for care quality and health system sustainability, as insufficient support can lead to caregiver burnout, increased healthcare utilisation, and poorer patient outcomes (22). Post-diagnostic support measures, including education, counselling, respite services, and structured care pathways, can empower caregivers and improve outcomes for people living with dementia (23).

Consultations indicated the importance of addressing structural inequalities affecting access to dementia care. **Migrant and ethnically diverse populations** often face cultural, linguistic, and systemic barriers that contribute to delayed diagnosis, limited access to services, and underutilisation of support resources (6, 8, 24, 25). These barriers may arise not only from individual cultural and linguistic needs but also from differences in the capacity of healthcare systems to provide culturally and linguistically appropriate dementia care, highlighting the importance of considering both structural and individual determinants of equitable access (26, 27). Dementia care research must therefore **consider intersecting factors** such as ethnicity, gender, sexuality, and socioeconomic status, which shape diverse experiences and may perpetuate inequities in access, quality, and outcomes of care (28, 29). Addressing these dimensions requires culturally sensitive and inclusive care.

Innovation is also shaping future dementia care systems. **Digital tools and artificial intelligence** offer potential solutions to workforce shortages and barriers to care access, but their implementation and scalability remain challenging. Digital interventions may improve access to care for people with dementia and their caregivers, yet adoption depends on usability, digital literacy, and sustained user engagement (30). Without addressing these factors, digital approaches risk reinforcing existing inequalities, particularly for rural, older, or disadvantaged populations (31, 32). Research should therefore evaluate digital interventions, sustainability models, and strategies to prevent digital exclusion (33).

Another priority was **strengthening resilience and adaptability within dementia care systems**. Diverse health system contexts, demographic change, and workforce pressures require care models capable of responding flexibly to evolving challenges while maintaining continuity and quality of care. Multiple actors in care provision further illustrate the need for adaptable care structures. These observations align with research on resilient dementia care systems in Europe (6, 21).

Finally, advances in **biomarkers and early diagnostic** technologies raise important ethical and psychological considerations. Early genetic and biomarker detection may generate uncertainty and distress, as biomarker results often indicate risk rather than a definitive diagnosis (34). Supportive care models providing clear information, counselling, timely consultation with appropriate specialists, education, and structured follow-up are needed to ensure responsible disclosure and help individuals and families manage uncertainty and stigma (34). Depending on individual needs and local care pathways, such consultations should involve dementia specialists with expertise in biomarker interpretation and may include neurologists, geriatricians, psychiatrists, genetic counsellors, specialist nurses, or multidisciplinary memory clinic teams (35).

### 3.4 Building an inclusive European research agenda

Beyond system-level challenges in dementia care, participants also emphasised the need to strengthen how dementia research is conceptualised, organised, and conducted across Europe. A European research agenda requires addressing conceptual, methodological, and collaborative dimensions shaping how dementia is understood and studied.

One important issue concerns how dementia is conceptualised in research and policy. Current European dementia policies often rely on **age-based models (65+)**, which may overlook younger people living with early-onset dementia. This reflects a structural limitation in how dementia is conceptualised and governed, as age thresholds often fail to capture the diversity of needs associated with cognitive impairment (36), potentially delaying access to diagnosis, care, and social protection (37). Need-based frameworks recognising dementia across the life course were therefore emphasised. Examples such as Denmark's integration of dementia and special-needs services illustrate how more flexible eligibility criteria may expand access and reduce inequities (38, 39). Future research should support policies addressing the full dementia spectrum and challenge age-based assumptions in policy, guidelines, and funding structures (37–39).

**Language and terminology** also play a critical role in shaping dementia research and policy. Participants highlighted that dementia-related terminology is not neutral: it influences public perceptions, service structures, and whose experiences are recognised (22, 40, 41). Terms used in policy and practice may reinforce stigma, define eligibility for services, and shape policy priorities (40, 41). Participants therefore emphasised the need for terminology that promotes inclusion rather than exclusion, for example shifting from terms such as “end of life” toward “palliative care” and from “dementia-friendly” to “dementia-inclusive.” This requires ongoing dialogue among researchers, practitioners, policymakers, and people with lived experience to develop inclusive and actionable terminology (22, 42).

Workshop discussions identified the importance of strengthening participatory approaches in dementia research. **Involving people with dementia in research design and decision-making** can improve the relevance, ethics, and impact of research. Alzheimer Europe similarly advocates the meaningful participation of people with dementia and caregivers as active contributors to research and policy (43). This requires attention to ethical, practical, and methodological issues, including informed consent, accessibility, and sustained engagement (44–46). Embedding participatory and co-production approaches within research frameworks can ensure that research priorities better reflect the lived experiences of people with dementia and their caregivers (44).

Another key dimension is **translating research into practice**. Participants emphasised that advancing dementia research requires stronger attention to implementation science, co-design with users, and sustainable funding mechanisms. Research outcomes alone are insufficient; uptake, feasibility, and long-term adoption are equally important. Evidence indicates that effective implementation depends on robust frameworks supporting feasibility and scalability (47), active engagement of patients, caregivers, and practitioners in co-designed interventions (48), and sustainable funding and infrastructure that support long-term impact (49, 50). Dedicated investment in implementation science is therefore essential to move frameworks and evidence-based interventions beyond conceptual development and into routine practice (47). This requires protected funding, appropriately trained personnel, implementation expertise, organisational leadership, and resources for adaptation, monitoring, and evaluation (51). Without such investment, even well-designed and co-produced frameworks risk remaining aspirational rather than producing sustained changes in clinical care, service delivery, and policy (47, 52).

Research infrastructures and conceptual alignment were also identified as priorities. Workshop discussions highlighted the need for **shared conceptual and methodological frameworks** capable of guiding dementia research across disciplines and national contexts. Limited alignment across countries and disciplines can hinder comparability and collaboration. Consensus-driven frameworks covering brain health, disability, ageing, and dementia progression could provide a common foundation for research agendas while facilitating interdisciplinary collaboration and translation of findings into policy and practice (53). Evidence from European research networks indicates that clearly defined frameworks improve collaboration, study comparability, and the applicability of research outcomes across diverse healthcare contexts (21, 53, 54).

Finally, **strengthening collaboration within European research systems** remained important. Cross-border collaboration, data sharing, and knowledge exchange are essential for dementia research. Examples such as Norway's research networks (NO-Age and NO-AD) illustrate how shared infrastructures can integrate regional expertise and support collaborative research (55). Despite policy support, challenges remain in data governance, funding, and coordination (22, 42). Strengthening collaborative structures can reduce fragmentation, improve comparability, and accelerate innovation across countries, supporting coordinated responses to dementia care challenges (56, 57).

Taken together, these priorities highlight the need to balance shared European approaches with adaptation to different healthcare, resource, cultural, and linguistic contexts. This need for both common principles and contextual flexibility informed the development of the EQUITABLE framework described below.

### 3.5 The EQUITABLE framework

The priorities identified across the consultation process highlighted the need for a common approach capable of addressing shared European challenges while remaining responsive to differences in healthcare systems, resources, cultural and linguistic contexts, and research capacity. Drawing on these findings, PANEUCARE moved from identifying individual priorities and collaboration gaps to developing a set of cross-cutting principles for a more equitable and participatory European dementia research landscape. Persistent fragmentation, uneven participation, and limited knowledge exchange across countries, disciplines, and stakeholders (3, 21), together with enduring East–West disparities in research participation, clinical trial access, and leadership roles (3, 58), underscore the need for more equitable and collaborative research structures. Integrating academic, clinical, and lived-experience knowledge through participatory research can improve the relevance, legitimacy, and uptake of research (42, 59, 60).

The consortium co-developed the EQUITABLE framework, outlining key dimensions for inclusive and context-sensitive dementia research:

- **E**quity in access to diagnosis, care and research participation
- **Q**uality focus
- **U**rgency of need, particularly in under-resourced settings
- **I**nvolvement of people with dementia and caregivers
- **T**ransdisciplinary collaboration
- **A**daptability and scalability
- **B**udget-consciousness and sustainability
- **L**inguistic, cultural, and contextual sensitivity
- **E**ngagement of stakeholders in all stages of research

These dimensions directly reflect the recurring priorities identified across the consultation process. Equity and urgency respond to disparities in access and the needs of under-resourced settings; quality reflects concerns regarding care standards and continuity; involvement and stakeholder engagement derive from the priorities expressed by people living with dementia and caregivers; transdisciplinary collaboration responds to fragmentation across health, social care, and research systems; adaptability, scalability, and budget-consciousness reflect variation in resources and implementation capacity across European contexts; and linguistic, cultural, and contextual sensitivity addresses barriers affecting migrant, minority, and other underserved populations.

Rather than operating independently, these dimensions are intended to be mutually reinforcing: equity and urgency help identify where needs are greatest, involvement and collaboration shape how priorities are defined, and adaptability, sustainability, and contextual sensitivity guide how these priorities can be translated across diverse European settings. The framework is therefore intended as a set of complementary guiding principles rather than a checklist to be applied uniformly, allowing their application to be adapted to the resources, priorities, and contextual characteristics of different European settings.

EQUITABLE shares principles with broader health-equity and responsible innovation frameworks, particularly their emphasis on equity, inclusion, participation, and responsiveness to societal needs (61, 62). Its specific contribution lies in translating these principles into a dementia-focused, pan-European framework derived from the combined perspectives of professionals, people living with dementia, and caregivers. In particular, EQUITABLE brings together regional disparities in research and care capacity, lived-experience involvement, contextual and linguistic sensitivity, and practical considerations of scalability and sustainability within a single set of guiding principles for European dementia research. Together, these principles aim to shift dementia research from short-term or project-based collaboration toward more sustainable and equitable pan-European research structures (63, 64).

Several practical actions were identified to support implementation. These include establishing EU-supported platforms for sustained cross-regional knowledge exchange to share and scale community-based and non-pharmacological dementia care innovations (21); creating structured co-creation mechanisms, such as regional research boards or thematic working groups involving people with dementia, caregivers, clinicians, and policymakers, to strengthen research relevance and uptake (43, 65); investing in capacity-building initiatives such as training, mentorship, and peer exchange to strengthen research capacity in underrepresented regions (66); investing in implementation science infrastructure and personnel to support local adaptation, evaluation, scale-up, and sustainability (64, 67); and promoting multi-country, multi-stakeholder governance within large European research consortia to improve equity in research leadership and cross-border collaboration (4, 68). These actions translate the EQUITABLE principles into more coordinated European dementia research structures (69–71).

**Figure 1:**
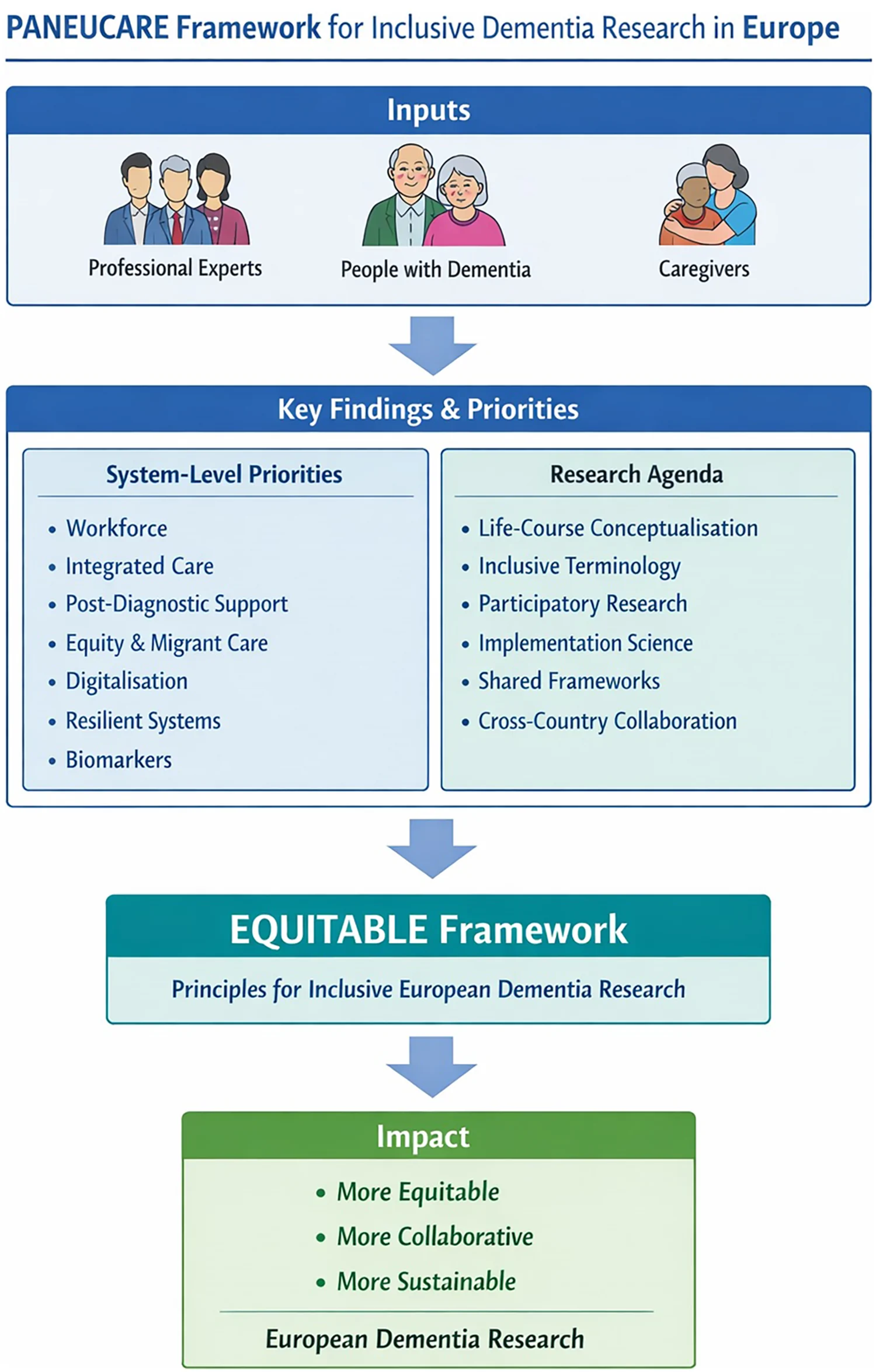
Conceptual overview of PANEUCARE findings and the EQUITABLE framework for inclusive dementia research in Europe.

Funding structures also shape participation in European dementia research. Current mechanisms often favour well-established institutions, leaving Southern and Eastern European regions underrepresented in large research programmes (58, 72). Inclusive funding architectures and capacity-building mechanisms may support broader regional participation. Alignment with the European Research Area (ERA) (73) and the Horizon Europe Widening Participation Strategy (74) could support more balanced participation across Europe. These interconnected findings and priorities are summarised in Figure 1.

## 4 Discussion

The PANEUCARE study highlights the complexity and diversity of dementia care across Europe, revealing shared challenges and context-specific innovations. Its contribution lies in combining professional expertise with lived experience from people with dementia and caregivers, offering a co-produced perspective linking research, practice, and policy. This approach moves beyond descriptive mapping of challenges and identifies actionable directions for equitable and context-sensitive dementia research.

A central insight is that regional diversity can be a strength rather than a limitation. While structural inequalities and resource constraints persist, particularly in Southern and Eastern Europe, these contexts also generate community-driven initiatives and adaptive care solutions. Such locally grounded innovations can complement more formalised systems in Western and Northern Europe. At the same time, the findings caution against interpreting European dementia care through fixed regional categories: substantial variation occurs within as well as between countries, and similar challenges may emerge under different organisational and resource conditions. These patterns highlight the potential for multidirectional cross-regional learning and collaboration rather than one-way transfer of stablished models, consistent with European policy frameworks promoting knowledge exchange and innovation in health research (13, 58, 73).

The inclusion of lived experience adds a critical dimension to this perspective. People with dementia and caregivers emphasised issues related to timely diagnosis, access to primary and specialist clinical care, post-diagnostic services, psychosocial and community-based support, information, caregiver resources, appropriately trained healthcare workers, meaningful participation, and culturally appropriate care. These dimensions are not always captured through professional expertise alone. This aligns with evidence that co-production in health research can improve both the relevance and implementation of interventions, shifting involvement from symbolic participation toward meaningful partnership in research governance (43, 44, 46).

Beyond meaningful participation, the findings also highlight an important cross-cultural dimension of equity. Migrant and minority ethnic populations may face linguistic and cultural barriers affecting access to services, communication, assessment, diagnosis, and the appropriateness of care pathways. Evidence on dementia assessment in culturally and linguistically diverse populations reinforces the need for cross-cultural competency that extends beyond translation to culturally responsive assessment and service provision (26, 27, 75).

Across the findings, four cross-cutting themes emerge: equity, sustainability, collaboration, and implementation. Persistent disparities in research participation, workforce capacity, and service access reflect broader structural inequalities in European health systems (5, 6, 21). At the same time, the recurrent gap between policy, research, and routine practice indicates that limited implementation is itself a major source of inequity (64). Addressing these challenges requires coordinated approaches that combine capacity building, equitable participation in research consortia, stronger cross-regional collaboration, and sustained investment in implementation science (63, 64). This includes dedicated funding, trained personnel, stakeholder involvement, context-sensitive adaptation, and evaluation of implementation processes and outcomes (51, 76). Such strategies are increasingly recognised as essential for improving research impact and policy uptake in complex health systems (77–79).

However, pursuing these priorities simultaneously also raises questions of feasibility and sustainability. Implementing priorities related to workforce capacity, specialist services, co-production, and sustained post-diagnostic support depends on financial and organisational resources that vary across European settings (80). Similarly, digital innovation may improve reach and partially alleviate workforce or geographical barriers, while at the same time excluding people with limited digital access, literacy, or support (81). Efforts to develop shared European frameworks create another tension: greater alignment can strengthen comparability and collaboration, but standardised approaches may require adaptation to differences in healthcare organisation, language, culture, resources, and implementation capacity (82). These tensions suggest that feasibility cannot be treated as a separate stage following priority setting; rather, resource requirements, distributional consequences, and contextual adaptability need to be considered when priorities are selected and implemented.

Within this context, the EQUITABLE framework provides a guiding structure for translating these insights into action, while recognising that its practical value depends on deliberate implementation strategies and sustained resourcing (83). By linking research priorities with principles of equity, collaboration, and contextual sensitivity, the framework offers guidance for strengthening inclusive and sustainable dementia research across Europe (20, 21).

Its practical applicability, however, will also depend on the structural conditions in which it is implemented. Politically, differences in national dementia strategies and policy priorities may affect the conditions for sustaining equity-oriented research agendas over time (80). Financially, institutions and regions with more limited research capacity may face greater difficulty securing the infrastructure, workforce, and long-term funding required for participation and implementation (80). Institutionally, fragmentation between health and social care and differences in governance arrangements may hinder transdisciplinary collaboration and translation into routine practice (80, 84). These conditions are unlikely to operate uniformly across countries, reinforcing the need for shared principles that can be adapted to different European settings.

Against this background, the value of EQUITABLE lies in providing a common structure through which implementation challenges can be considered when research priorities, partnerships, and strategies are designed. The next step is to move from framework development to prospective application and evaluation, examining its feasibility and acceptability across European contexts and whether its use can strengthen participation, implementation, and equity in dementia research.

### 4.1 Strengths and limitations

A strength of this Perspective is its iterative three-phase consultation process, which brought professional and lived-experience perspectives into the identification and refinement of research priorities. This approach enabled priorities emerging from different stakeholder groups to be considered together rather than in isolation. Several limitations should nevertheless be acknowledged. Professional participants were invited based on relevant expertise and geographical diversity, while lived-experience participants were members of established European advisory groups. The sample was not intended to be statistically or population-level representative and may not reflect perspectives beyond those represented in the consultation, particularly among people less connected to research or advocacy networks. Workshop findings may also have been influenced by participants’ backgrounds, experiences, and group dynamics; therefore, the resulting synthesis should not be interpreted as a formal European consensus. Finally, EQUITABLE emerged from this consultative process and has not yet been prospectively evaluated. Its feasibility and added value will need to be examined through application in different European settings, including how the framework can be adapted to local implementation conditions.

## 5 Conclusion

The PANEUCARE study highlights diverse dementia care realities across Europe, revealing shared challenges alongside context-specific innovations. Integrating professional expertise with lived experience can strengthen the relevance of dementia research and policy, while regional diversity provides opportunities for multidirectional learning across European contexts. The EQUITABLE framework provides guiding principles for embedding equity, participation, collaboration, and contextual sensitivity within European dementia research, but its practical value will depend on context-specific implementation and evaluation. Immediate next steps should therefore focus on applying and evaluating the EQUITABLE principles across diverse European health and research contexts, developing indicators to assess their feasibility and impact, and broadening participation to include populations and regions that remain underrepresented in dementia research. Such efforts will require sustained collaboration among researchers, people living with dementia, caregivers, practitioners, policymakers, and funders, together with attention to the structural conditions that influence implementation

## Data Availability

The original contributions presented in the study are included in the article/Supplementary Material, further inquiries can be directed to the corresponding author.
